# Children with Severe Malnutrition: Can Those at Highest Risk of Death Be Identified with the WHO Protocol?

**DOI:** 10.1371/journal.pmed.0030500

**Published:** 2006-12-26

**Authors:** Kathryn Maitland, James A Berkley, Mohammed Shebbe, Norbert Peshu, Michael English, Charles R. J. C Newton

**Affiliations:** 1 Centre for Geographic Medicine Research (Coast), Kenya Medical Research Institute, Kilifi, Kenya; 2 Department of Paediatrics, Faculty of Medicine, Imperial College, London, United Kingdom; 3 Wellcome Trust Centre for Clinical Tropical Medicine, Imperial College, London, United Kingdom; 4 Centre for Clinical Vaccinology and Tropical Medicine, Churchill Hospital, University of Oxford, Oxford, United Kingdom; 5 Department of Paediatrics, John Radcliffe Hospital, University of Oxford, Oxford, United Kingdom; 6 Neurosciences Unit, Institute of Child Health, The Wolfson Centre, Mecklenburgh Square, London, United Kingdom; College of Medicine, Malawi

## Abstract

**Background:**

With strict adherence to international recommended treatment guidelines, the case fatality for severe malnutrition ought to be less than 5%. In African hospitals, fatality rates of 20% are common and are often attributed to poor training and faulty case management. Improving outcome will depend upon the identification of those at greatest risk and targeting limited health resources. We retrospectively examined the major risk factors associated with early (<48 h) and late in-hospital death in children with severe malnutrition with the aim of identifying admission features that could distinguish a high-risk group in relation to the World Health Organization (WHO) guidelines.

**Methods and Findings:**

Of 920 children in the study, 176 (19%) died, with 59 (33%) deaths occurring within 48 h of admission. Bacteraemia complicated 27% of all deaths: 52% died before 48 h despite 85% in vitro antibiotic susceptibility of cultured organisms. The sensitivity, specificity, and likelihood ratio of the WHO-recommended “danger signs” (lethargy, hypothermia, or hypoglycaemia) to predict early mortality was 52%, 84%, and 3.4% (95% confidence interval [CI] = 2.2 to 5.1), respectively. In addition, four bedside features were associated with early case fatality: bradycardia, capillary refill time greater than 2 s, weak pulse volume, and impaired consciousness level; the presence of two or more features was associated with an odds ratio of 9.6 (95% CI = 4.8 to 19) for early fatality (*p* < 0.0001). Conversely, the group of children without any of these seven features, or signs of dehydration, severe acidosis, or electrolyte derangements, had a low fatality (7%).

**Conclusions:**

Formal assessment of these features as emergency signs to improve triage and to rationalize manpower resources toward the high-risk groups is required. In addition, basic clinical research is necessary to identify and test appropriate supportive treatments.

## Abstract 2

A retrospective examination of major risk factors associated with in-hospital deaths in children with severe malnutrition has identified admission features that could help distinguish those at highest risk.

## Editors' Summary

**Background.:**

Severe malnutrition is thought to be responsible, at least in part, for a large proportion of the many millions of deaths every year among children below the age of five years. The World Health Organization (WHO) has developed guidelines for management of the severely malnourished child in the hospital. These guidelines outline ten initial steps for routine care, followed by treatment of associated conditions and rehabilitation. However, death rates among children admitted to hospital with severe malnutrition are worryingly high, commonly 20% or sometimes even higher. Many hospitals have reported that following introduction of the WHO guidelines, the death rates have been cut, but not to a level that the WHO defines as acceptable (5% or lower).

**Why Was This Study Done?:**

In the region where this study was done, an area on the coast of Kenya, East Africa, malnutrition is very common. The local hospital, Kilifi District Hospital, currently reports a death rate of approximately 19% among children admitted with severe malnutrition, even with implementation of the WHO guidelines. A group of researchers based at the hospital wanted to see if they could identify those children who were most likely to die. Their aim was to see which aspects of the children's medical condition put them at highest risk. This information would be useful in ensuring that high-risk children received the most appropriate care.

**What Did the Researchers Do and Find?:**

The researchers studied all severely malnourished children over three months of age who were admitted to the Kilifi District Hospital between September 2000 and June 2002. The children were treated according to the WHO guidelines, and the research group collected data on the condition of the children after treatment (their “outcomes”), as well as for relevant clinical signs and symptoms. The study involved 920 children, of whom 176 died in hospital (a death rate of 19%). They then examined the data to see which characteristics on admission were associated with early death (less than 48 h) and later deaths. They found that four clinical features, which could be easily ascertained at the bedside on admission, were associated with a large proportion of the early deaths. These four signs were slow heart rate, weak pulse volume, depressed consciousness level, and a delayed capillary refilling time (as tested by pressing a fingernail bed to blanche the finger, releasing it, and observing the time taken to reperfuse the capillaries—or recolor the nailbed). The researchers proposed that these findings, together with a number of other features that were associated with the later deaths could be used to identify three groups of patients differing in their need for emergency care: a high-risk group (with any of the four signs listed above, or hypoglycemia, and among whom mortality was 34%); a moderate-risk group (among whom mortality was 23%); and a low-risk group (mortality 7%).

**What Do These Findings Mean?:**

First, the death rate amongst these children was very high even though WHO guidelines were used to guide management. The signs reported here as indicators of poor outcome may prove useful in future in identifying high-risk individuals to ensure they receive the right treatment. However, the indicators proposed here would need further evaluation before current guidelines for treatment of the severely malnourished child could be changed.

**Additional Information.:**

Please access these Web sites via the online version of this summary at http://dx.doi.org/10.1371/journal.pmed.0030500.

• Information on severe malnutrition is available from the World Health Organization

• Management guidelines from the WHO can also be downloaded in many languages

• UNICEF, the United Nations Children's Fund, provides relevant resources and statistics as well as information about its programs addressing malnutrition worldwide

• Information from Médecins Sans Frontières (MSF) on acute malnutrition worldwide and MSF's response to current emergencies

## Introduction

**Figure 1:**
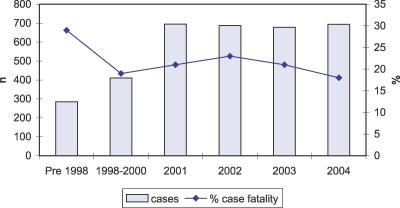
Annual Admissions of Children with Severe Malnutrition and Case Fatality Rates Prior to 2000, malnutrition was defined as weight for age (WAZ) less than −4 or oedematous malnutrition. Annual admissions rates: 1991–1998 (3,800), 1998–2000 (4,877), 2001 (5,136), 2002 (4,878), 2003 (5,583), and 2004 (5,004).

The World Health Organization (WHO) regards as unacceptable a mortality rate of over 20% in children with severe malnutrition, a situation that is common in many hospitals in sub-Saharan Africa [1,2]. These high case fatality rates are often attributed to insufficient staff training and poor compliance with the recommended protocol, resulting in faulty case management [3–8]. The WHO has developed consensus management guidelines and suggests that, with strict protocol adherence, mortality should be less than 5% [2]. The management guidelines includes a stabilisation phase in which life-threatening problems are identified and treated, a staged introduction of milk-based nutritional rehabilitation, micronutrient and vitamin supplementation, and empiric use of antimicrobial and antihelminthic treatment. Over the last decade, some centres have reported reduced case fatality rates by strict compliance to the treatment recommendations [9,10]. Nevertheless, two studies conducted in African hospitals examining the feasibility and sustainability of WHO severe malnutrition management guidelines have reported that inpatient case fatality rates of up to 46% had been reduced to 18%, at best, following implementation [3,6]. In these and other studies, failure to further improve outcome has been variously ascribed to HIV infection, lack of maternal participation in the feeding program, inadequate care and prescription errors, and overprescription of intravenous therapies and blood transfusion [6,11,12]. At our hospital, we too found that fatality rates were cut to only 19% from over 30%, after the implementation of the WHO guidelines (Figure 1). Comparable with the experience at other centres [9], we too found that death commonly occurs during the first 48 h after hospital admission. We therefore reasoned that children at high risk of early mortality may not be adequately identified at admission by the current WHO protocol. In order to determine the major associations with poor outcome, especially for those dying early after admission—a group that could be identified and targeted for appropriate treatment—we undertook a review of children admitted with severe malnutrition.

## Methods

### Study Site and Population

The study was conducted at Kilifi District Hospital, situated on the coast of Kenya. The hospital serves a rural population of over 230,000, 85% of whom are subsistence farmers and only 4% are estimated to have waged employment. The population density is high in relation to the agricultural potential, rendering Kilifi District the second poorest in Kenya in terms of per capita income. The district also has one of the lowest female literacy rates [13]. Maize is the staple food. Malnutrition is endemic within the community; over 40% of children less than 5 y old have anthropometric features of undernutrition [14], and 46% have biochemical markers of iron deficiency [15]. The paediatric ward at Kilifi District Hospital admits more than 5,000 children each year, with severe malnutrition being the fourth commonest cause of admission to hospital and second commonest cause of in-hospital fatality. In contrast to the Indian subcontinent, cholera, typhoid, and dysentery are uncommon [16]. Distinct from other cohorts in Africa, the overall HIV prevalence amongst children admitted to hospital at the time of the study was only 8% [16]. Staffing levels on the paediatric ward are low, as in most African hospitals; typically, two to three nurses care for up to 120 children, including neonates.

### Data Collection

Government-employed clinical officers, separate from the study team, referred children from the outpatient department to the paediatric ward. All children admitted to the ward were assessed by a member of the clinical research team. The children were weighed (Soehnle model 7300; CMS Instruments, London, United Kingdom) and had their length (or height) and mid–upper arm circumference (MUAC) measured using standard equipment [17,18]. A weight-for-height z-score (WHZ) was calculated for each individual by using Epi Info v2000 (Centers for Disease Control and Prevention, Atlanta, Georgia, United States). For this study, severe malnutrition was defined as either (1) visible, severe wasting [19] (where height/length data were not available), (2) visible, severe wasting plus WHZ less than −3, (3) MUAC less than 11.5 cm to define marasmus, or (4) symmetrical oedema involving at least the feet to define oedematous malnutrition (kwashiorkor), irrespective of WHZ score or MUAC [17]. Oxygen saturation and heart rate were recorded by a pulse oximeter (Nellcor [http://www.nellcor.com]); respiratory rate and characteristics were determined by the medical staff. At the time of the study, facilities for routine counselling and HIV testing were limited, and antiretroviral therapy was not in routine use. Blood cultures were processed by a BACTEC 9050 system instrument (Becton Dickinson [http://www.bd.com]), and antibiotic susceptibilities were determined at the end of the study using E-test (AB Biodisk [http://www.abbiodisk.com]) [16,20]. Parents or guardians of individual study participants gave written informed consent in their own language to be part of the routine clinical surveillance. Data prospectively collected from all paediatric admissions have been described elsewhere [21].

### Standard Management of Severe Malnutrition

Children with severe malnutrition were treated according to WHO guidelines insofar as staffing levels allowed [2]. Children with hypothermia (axillary temperature <35 °C) were placed under a blanket plus a radiant light; hypoglycaemia (blood glucose <3 mmol/l) was treated with 5 ml/kg of 10% dextrose intravenously followed by 50 ml of 10% dextrose via nasogastric tube. Malnutrition oral rehydration solution (ReSoMal) [2] was given to children with significant diarrhoea (more than three watery stools/day). Intravenous fluids were reserved for children showing evidence of decompensated shock, defined as weak/impalpable pulses with impaired consciousness. Data were not systematically collected on the number receiving ReSoMal or the intravenous fluids (or volumes administered). Blood transfusions, 10 ml/kg whole blood given slowly over 3–4 h, were reserved for children with symptomatic severe anaemia (haemoglobin [Hb] <5 dg/l) and those with decompensated shock. Vitamin A, multivitamin supplements, mineral mix, potassium chloride (6 mmol/kg/d), and folic acid 5 mg were given daily until discharge [2]. Iron supplements were given to children after clinical improvement and resolution of oedema. All children received intravenous ampicillin (50 mg/kg four times a day) and intramuscular gentamicin (7.5 mg/kg once daily) for at least 5 d. Chloramphenicol and ceftriaxone were used as second-line antimicrobials or when indicated by microbiological results. In addition, children received mebendazole 100 mg 12-hourly for 3 d and metronidazole 5 mg/kg 8-hourly for 5 d. Malaria infection was treated with oral sulfadoxine-pyrimethamine or by intravenous quinine for those unable to take oral medication.

### Feeding

For children reluctant to take feeds, a nasogastric tube was fixed for milk feeding. F75 and F100 milk formulae were prepared according to standard protocols [2]. Initially, F75 was given at a rate of 130 ml/kg/d (every 3–4 h), based upon admission weight; then the F75 formula was gradually replaced by F100 (in accordance with WHO guidelines [2]) as soon as appetite returned and oedema started to resolve. Thereafter, the volume of milk was calculated based on daily weights. In August 2001, during an intensive period of temperature monitoring, a blanket was added to the treatment chart, and a dedicated on-ward “milk kitchen” staffed by trained workers to supply warm milk feeds was instituted.

### Analysis

We analyzed data from children aged greater than 3 mo who were admitted with severe malnutrition between 01 September 2000 and 30 June 2002. Dichotomous and categorical variables were created from continuous variables. Binary variables were created from admission measures and from cut-offs defined by levels that would imply a definite need for urgent therapeutic intervention (American Pediatric Advanced Life Support [PALS] guidelines [22]) or outside the 95% confidence interval (CI) of the normal distribution (see Table 1). Separate analyses compared the characteristics of survivors with early deaths (≤48 h) and late deaths (>48 h). A 48-h time point was chosen in order to provide both some comparable uniformity across the literature for in-hospital mortality [9,23] and a period that was likely to be strongly influenced by the treatment decisions made by the admitting medical team. Likelihood ratios (LR) (95% CI) were used to examine the likelihood of a clinical variable predicting early death or late death. Multivariate logistic regression was used to estimate the relative contribution of each feature to early deaths. We recognize that, when there are a large number of independent variables to choose from, the problem of variable selection arises. Although logistic regression has been widely used in our previous publications [16,17,24,25], standard methods for logistic regression only consider bivariate factors (present or absent), and thus have been previously criticized as yielding *p-*values that are too low and standard errors that are too small, as well as for other reasons [26]. We nonetheless adopted logistic regression here because of its familiarity, and because the resulting model makes substantive sense. The indicator variables for each factor were values that are recognized as lying within the normal range. Receiver operator characteristic curve analysis was used to assess the ability of the four features identified by regression analysis (scored between 0 [no features present] to 4 [all four present]) to predict a fatal outcome. Analysis was performed using SPSS V13 (SPSS [http://www.spss.com]) and Stata V8 (Timberlake [http://www.timberlake.co.uk]).

**Table 1:**
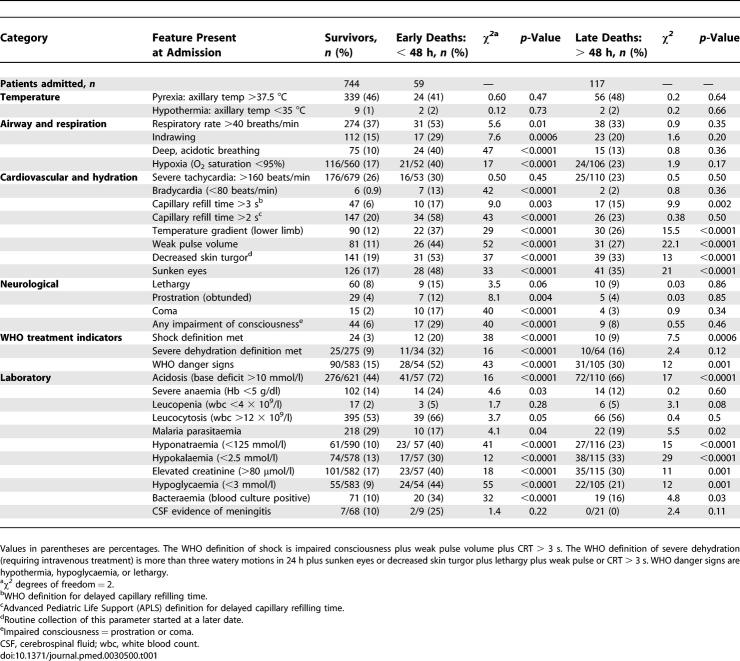
Clinical Features in Survivors and Early and Late Fatalities

## Results

**Figure 2:**
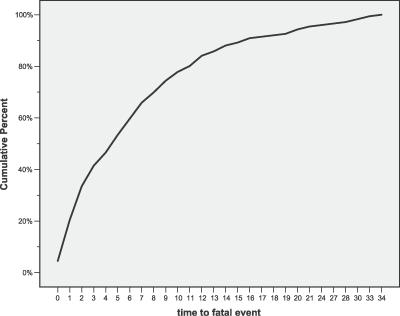
Timing of In-Hospital Death Day 14 represents the percentage of all deaths (176) that occurred in the second week (day 14 to 20), and day 21 represents the percentage of all deaths that occurred on or after 21 d of inpatient treatment.

Of 7,869 admissions of children aged over 3 mo during the period of the study, 920 (12%) fulfilled the criteria for severe malnutrition. Oedematous malnutrition was present in 42%. Males constituted 52% of the children; the median age was 25 mo (interquartile range [IQR] 16 to 46), children with oedema being slightly older (29 mo [IQR 19–52]). Overall, 176 (19%) children died during hospital stay. Compared with those with oedematous malnutrition alone and oedema complicated by marasmus, mortality was greatest in children presenting with marasmus (χ^2^ = 17.5, *df* = 2, *p <* 0.0001). Of those dying, 59 (33%) died within 48 h and 73 (41%) died within 72 h of admission (see Figure 2). Comorbidities such as gastroenteritis (16%), malaria (8%), anaemia (12%), pneumonia (8%), laboratory-confirmed bacteraemia (30%), and other invasive bacterial diseases (meningitis and osteomyelitis) (4%) complicated the early deaths. In the 117 children dying after 48 h, gastroenteritis (26%), bacteraemia (17%), pneumonia (7%), other invasive bacterial diseases (5%), and chronic diseases such as tuberculosis (3%), known HIV infection (5%), renal failure, and neurological impairment were documented as contributory.

### Clinical Signs

Clinical features associated with fatal outcome are presented in Table 1. In general, the frequencies of abnormal triage parameters were more common in children who died compared to those who survived. For most parameters, the frequencies did not vary between the anthropometric groups. Recorded pyrexia on admission was common (>45%); conversely, hypothermia was rare, even in the early mortality group. The emergency signs of shock (defined as weak/impalpable pulses with impaired consciousness) were present in 20% of early deaths (LR = 6.3 [95% CI = 3.1 to 12.6]). The sensitivity and specificity of one or more of the “danger signs” suggested by WHO (lethargy, hypothermia, or hypoglycaemia) to predict early mortality was 52% and 84%, respectively; positive LR = 3.4 [95% CI = 2.2 to 5.1). Markers that provided little prognostic information included Hb, white cell count, and malaria parasitaemia. Visible cyanosis (one of the Integrated Management of Childhood Illness emergency signs [27]) was uncommon; only 14 (1.5%) were assessed as cyanosed at admission, of which four died (29%).

### Early and Late Deaths

Hypoxia and increased work of breathing, principally deep (acidotic or Kussmaul) respiration at admission were more common in early deaths; positive LR for predicting early death = 4.8 (95% CI = 2.8 to 8.0) compared to LR = 1.3 (95% CI = 0.8 to 2.3) for late death. Although tachycardia had no prognostic significance, bradycardia was highly specific for early mortality; LR = 13.0 (95% CI = 4.7 to 36). In this group, bradycardia did not appear to be a pre-terminal event: only two deaths occurred within 24 h of admission: the other five children died between 24 and 48 h after admission. Over 50% of children with bradycardia had features of multisystem involvement including seizures, weak pulse, deep breathing, impaired consciousness, severe acidosis, hypokalaemia, or elevated creatinine levels.

Both delayed capillary refill time (CRT) (>3 s) and temperature gradient (cold extremities compared to warmer proximal limbs, assessed by running the back of the hand *up* the shin) were more common among children who died, but did not discriminate between early or late deaths. A less-stringent value of delayed CRT of more than 2 s (rather than CRT >3 s) had a much better prognostic value for early death; positive LR = 2.8 (95% CI = 2.0 to 4.1). Similarly, weak pulse volume was also a good marker of early mortality; positive LR = 3.0 (95% CI = 2.0 to 4.6). Lethargy was common in all groups and did not discriminate well between survivors and those who died; LR for predicting early death = 2.0 (95% CI = 1.0 to 4.0). Conversely, depressed consciousness level (prostration or coma) was more frequent in early deaths and was highly predictive of early mortality; positive LR = 4.7 (95% CI = 2.7 to 8.1). Severely deranged biochemical values were common in all cases; but hypoglycaemia, hyponatraemia, and severe acidosis (base deficit > 10 mmol/l) were all associated with early case fatality. For hypoglycaemia, the LR for identifying early deaths = 4.0 (95% CI = 2.5 to 6.3) and for late deaths, LR = 2.2 (95% CI = 1.4 to 3.6). Hypokalaemia was more common in the late deaths (LR = 2.6 [95% CI = 1.8 to 3.8]), and was more frequent in children with marasmus (78/354 [22%]) and oedematous marasmus (35/204 [17%]) than kwashiorkor alone (16/192 [8%]; χ^2^ = 16; *p <* 0.0001).

### Dehydration

**Figure 3:**
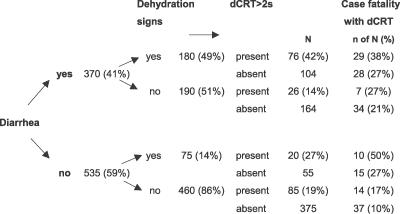
Value of the Signs of Dehydration and Delayed Capillary Refill Time (dCRT) in 905 Severely Malnourished Children with and without Diarrhoea Diarrhoea is defined as more than three watery motions per 24 hours. “Dehydration signs” are sunken eyes and/or decreased skin turgor.

A total of 153/211 (73%) children with decreased skin turgor and 146/196 (74%) of children with sunken eyes had a recent history of diarrhoea (more than three watery stools/day). The difficulty of differentiating the signs of acute dehydration from chronic features of malnutrition is recognized [2]. We therefore examined whether the features of acute dehydration (sunken eyes or decreased skin turgor) had some value in identifying high-risk groups in children with and without acute diarrhoea (Figure 3). Overall, 225 were assessed to have features of dehydration, 180 (71%) of these were in children presenting with acute diarrhoea (χ^2^ = 132; *p <* 0.001). In the group with diarrhoea, 98 (26%) died. Dehydration was of limited use as a prognostic sign because only 57 (58%) of children who died were assessed as “dehydrated” (χ^2^ = 5.2; *df* = 1; *p* = 0.02). However, the addition of delayed CRT > 2 s was found to delineate a group with a high mortality (38%), and may have some utility for rationalizing resources or treatments; LR = 1.3 (95% CI = 0.84 to 2.1). For the group without a history of diarrhoea, apparent features of dehydration and delayed CRT were ominous; 50% of these children subsequently died (LR = 6.5 [95% CI = 2.7 to 15.6]). In the 375 (41%) children without diarrhoea, signs of dehydration, or a delayed CRT > 2 s, mortality was much lower (10%).

### Intravenous Therapies

Data were not systematically collected on intravenous fluids administration, so their effect on mortality cannot be reported. In general, WHO guidelines were followed, and intravenous therapies were reserved for children experiencing complications of shock, including severe dehydration. Mortality in those receiving a whole blood transfusion, 35/176 (20%), was similar to the nontransfused group: 133/744 (18%); *p* = 0.54. Mean admission Hb in the severely anaemic group was 3.5 g/dl (standard error [SE] = 0.08). Blood transfusions were prescribed more frequently in the early fatality group (17/59; 29%) than the in late fatality group (8/117; 15%) or survivors (133/744; 15%); *p* = 0.08. The main indication for transfusion in the early fatality group was hypovolemic shock (14/17; 82%) rather than for the treatment of severe anaemia. A number of children admitted with Hb > 5g/dl subsequently required a transfusion for the subsequent development of severe anaemia (Hb < 5 g/dl): 50% of these had Plasmodium falciparum parasitaemia. Mean admission Hb (7.4 g/dl [SE = 0.19]) in the parasitaemic subgroup was lower than the parasitaemic group that was not subsequently transfused; Hb = 9.0 g/dl (SE 0.18); *p <* 0.0001.

### Multivariate Analysis

Risk factors predicting early mortality (*p <* 0.05) that were identified by univariate analysis and that could be readily ascertained at the bedside were entered into a regression model. Four admission features were associated with early case fatality: (1) bradycardia (odds ratio [OR] = 8.8 [95% CI = 2.6 to 30]; *p* = 0.001), (2) CRT > 2 s (OR = 4.1 [95% CI = 2.1 to 8.0]; *p <* 0.0001), (3) weak pulse volume (OR = 2.3 [95% CI = 1.1 to 4.7]; *p <* 0.02), and (4) impaired consciousness level (prostration or coma) (OR = 2.6 [95% CI = 1.2 to 5.6]; *p* = 0.02). Receiver operator curve analysis of the presence of these four parameters to identify those at risk of early death was 0.79 (95% CI = 0.72 to 0.86). A prognostic score of two or more of these variables was associated with early death (OR = 9.6 [95% CI = 4.8 to19]; *p <* 0.0001) compared to those without these variables. In the early death group, a score of one or more was present in 16/19 (84%) of those with bacteraemia and 28/33 (85%) of those with signs of dehydration. Hypoglycaemia was present in 18% of children with a score of one or two, and 33% of those with scores of three or four.

These findings were used to delineate three groups that differed in prognosis and the need for emergency care, which could be used to prioritize treatment and routine surveillance (Box 1). A very high–risk group was identified that included children with any one of the prognostic variables or hypoglycaemia, for which case fatality was 94/277 (34%), compared to 54/423 (12%) without any of these features (χ^2^ = 45.0; *p <* 0.0001). Children without high-risk features were further resolved into a moderate-risk group (with any one of the features of deep acidotic breathing, acute dehydration, lethargy, hyponatraemia, or hypokalaemia), with an attendant fatality of 32/106 (23%), and a low-risk group without any of the above features, in which only 22/285 (7%) died.

**Box 1: Proposed Triage System for Children with Severe Malnutrition to Improve Identification of High-Risk Groups for Targeting of Health Care in Resource-Limited Settings**

**High Risk**

Immediate risk of early death and greatest requirement for close observation and monitoring

- Depressed conscious state

  ○ Prostration (inability to sit up) *or*
  ○ Coma (inability to localize a painful stimulus)

- Bradycardia (heart rate < 80 beats per minute)
- Evidence of shock with or without dehydration (see below)

  ○ Capillary refill time ≥ 2 s *or*
  ○ Temperature gradient
  ○ Weak pulse volume

- Hypoglycaemia < 3 mmol/l

**Moderate Risk**

Need for close supervision

- Deep acidotic breathing
- Signs of dehydration (plus diarrhoea: > 3 watery motions/24 h)

  ○ Sunken eyes *or*
  ○ Decreased skin turgor

- Lethargy
- Hyponatraemia (sodium < 125 mmol/l)
- Hypokalaemia (potassium < 2.5 mmol/l)

**Low Risk**

Limited requirement for close supervision

- None of the above

Note: In-hospital mortality in the three groups at Kilifi were as follows: high-risk group, 34%; moderate-risk, 23%; and low-risk, 7%.

### Bacteraemia at Admission

Laboratory-confirmed bacteraemia occurred in 110 (12%) children: 39 (36%) died compared with 37/810 (17%) deaths in those without bacteraemia (χ^2^ = 21.5; *p <* 0.0001). Overall, proven invasive bacterial disease at admission complicated 23% of all deaths: 52% of these fatalities occurred within 48 h of admission. In the early mortality group, ten (17%) children had gram-positive organisms, 13 (22%) had gram-negative organisms, and four had mixed infections. Of those dying more than 48 h after admission, eight (8%) had gram-negative organisms and nine (9%) had gram-positive organisms compared to 29 (4%) and 37 (5%) of the survivors, respectively. In 60% of cases, three main organisms were responsible for invasive bacterial disease: Streptococcus pneumoniae (*n* = 38; 35%), Escherichia coli (*n* = 13; 12%), and nontyphoidal salmonellae (NTS) (*n* = 11; 10%). Other principle isolates included Haemophilus influenzae (*n* = 9; 8%), Staphylococcus aureus (*n* = 9; 8%), and Streptococcus spp. (A, B, and D) (*n* = 10; 9%). E. coli (4; 18%) and Pseudomonas aeruginosa (4; 18%) bacteraemias were more commonly associated with early mortality, whereas NTS were the more frequent cause of gram-negative infection in later deaths (18%) and in survivors (10%).

### Admission Blood Cultures: Sensitivity of Organisms

In vitro sensitivity data suggest that the ampicillin and gentamicin first-line combination provided optimal antimicrobial cover: 63/74 (85%) isolates were fully sensitive to the combination. Antimicrobial resistance was not associated with early deaths: 15/17 (88%) of isolates were sensitive to the combination in vitro, which compared favourably to 38/42 (90.5%) in survivors; *p* = 0.79). Antimicrobial resistance of the admission isolates was, however, associated with late deaths (>48 h); 10/15 (67%) (χ^2^ = 4.7; *p* = 0.03). Addition of chloramphenicol (the WHO-recommended second-line agent) would not have improved antimicrobial coverage, as this would have increased the number of sensitive isolates by only one (1/55; 1.8%).

### Nonadmission Blood Cultures

A total of 11 (9%) of the 117 late deaths had a positive blood culture, taken after admission, increasing the proportion of deaths associated with a proven invasive bacterial disease to 24% for late deaths and to 26% for all deaths. There was evidence to suggest that antimicrobial resistance may have been contributory to the later deaths: only 4/11 (36%) of the nonadmission bacteraemias in this group were sensitive to the ampicillin/gentamicin combination. Of those tested against cefotaxime and chloramphenicol, 3/8 (38%) and 6/12 (50%) were fully sensitive, respectively. Although ciprofloxacin was not in routine use at the time of the study, 10/11 (91%) nonadmission isolates were fully sensitive in vitro.

## Discussion

In a large case series of severe malnutrition presenting to a district hospital in Kenya, managed according to WHO protocol insofar as staffing levels permitted, case fatality was 19%. Over 30% of these deaths occurred within 48 h of admission. Sepsis and invasive bacterial disease were a major cofactor in many deaths; more specifically, bacteraemia, identified by blood culture, was present in 27% of all deaths, 37% of early deaths, and 25% of late deaths. The sensitivity and specificity of one or more of the recommended dangers signs suggested by the WHO (lethargy, hypothermia, or hypoglycaemia) to predict early mortality were 52% and 84%, respectively. In addition, we found that four clinical features, readily assessable at the bedside—bradycardia, a CRT > 2 s (a less-stringent value than currently advocated), weak pulse volume, and impaired consciousness level (defined as prostration or coma)—were present in over 80% of children dying early, and may identify those at greatest risk. Three out of four of these signs are included in the Emergency Triage Assessment and Treatment (ETAT) system designed for general paediatric triage [28]. Hypoglycaemia complicated 18% of cases with one to two of these features, and affected an even greater proportion (33%) if three or four of these prognostic features were present.

**Figure 4:**
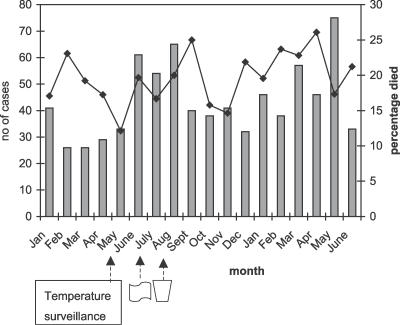
Monthly Admission of Children with Severe Malnutrition (Bars) and Percentage Case Fatality (Line) for 2001–2002 In July 2001, during a period of 6-h temperature surveillance, the routine prescription of a blanket (see blanket symbol) was added to the treatment chart, and in August 2001 (see cup symbol), F75 and F100 were prepared (day and night) in a specifically devoted “milk kitchen” with dedicated trained workers to expressly supply the malnourished with warm milk feeds every 4 h, or as required.

Why, when put into practice at our hospital, did current management guidelines fail to reduce the mortality to the recommended value of less than 10%? Was implementation of standard management protocols limited by inadequate training and poor adherence to the management protocol? Or did the context of a busy paediatric ward constrain the practicality of the current protocol by a disproportionately large patient-to-health personnel ratio and inefficient use of the limited manpower resources? A recent paper by English and colleagues [29] highlights the lack of capacity, training, and consumable resources at most district hospitals across Kenya. Of the hospitals surveyed, only 35% recorded weight, and 78% had no guidelines for the management of severe malnutrition [30]. In contrast, care given to children at our hospital, despite the limitations of staffing, is directed by protocol and reinforced by regular training. Clearly illustrated (see Figure 4) are the limitations, in terms of reduction in mortality, that are achieved by a period of training followed by increased monitoring, a secure provision of warm milk, and an intensive period of temperature surveillance. Failure of these to significantly impact upon mortality suggests a more complex aetiology, and was one factor motivating this retrospective review.

Increased awareness and uptake of the WHO guidelines through simple procedures [17] to tailor certain aspects of care such as triage and pragmatic allocation of resources may improve their implementation. Our approach to investigating factors associated with early mortality included examining the utility of the current paediatric emergency triage guidelines (American PALS) [22]. Similarly, more relevant emergency triage assessments (ETAT) have been evaluated and are currently recommended for the seriously ill children presenting in resource-poor countries [28]. Nevertheless, for the diagnosis of shock the presence three features are required (cold feet or hands, weak and fast pulse), which have resulted in limited numbers identified for which fatal outcome is almost universal [28]. Our findings included a number of parameters including those associated with shock, which can be easily identified with minimal training, to delineate a high-risk group to target emergency treatment and monitoring (Box 1). An example of this type of approach to managing diarrhoea and/or signs of acute diarrhoea is shown in Figure 3, with which it may be possible to rationalize resources for surveillance and make decisions about using intravenous therapies based upon the presence of a delayed CRT. However, owing to the limitations of the current study and the concern that escalating the number of intravenous infusions may have detrimental consequences, further clinical research is required to prospectively validate these findings before advocating any change to current treatment guidelines.

The triage parameters associated with early mortality suggest that many children had cardinal features of hypovolemic shock including bradycardia, weak pulse volume, deep acidotic breathing, impaired consciousness, and hypoglycaemia [31]. These signs taken together with the high prevalence of invasive bacterial disease in both early and late deaths in our population and that of others [7,32,33] suggest that acute sepsis plays a major role in these deaths. Furthermore, improved outcome will depend upon early identification of those at risk, better antimicrobial therapy, and targeted supportive treatments. The urgent correction of intravascular volume deficits by rapid volume expansion management using isotonic crystalloidal or colloidal solutions is central to contemporary paediatric critical care practice and would seem a logical treatment for children with severe malnutrition presenting with shock. Nevertheless, current guidelines do not include either of these solutions [34], instead, recommending hypotonic solutions (half-strength Darrows or 50% Ringer's lactate) because of concerns over the risk of sodium and fluid overload that are peculiar to this patient group. At present, the data supporting either approach in children with severe malnutrition are imperfect; the balance of risk and benefit can only be resolved with certainty by definitive clinical trials. Given the unacceptably high mortality rates suggested by our studies, such trials are urgently needed.

Our data suggest that the current regime of ampicillin and gentamicin is appropriate as first-line therapy, with up to 90% of isolates being fully sensitive even in children dying less than 48 h after admission. Noorani et al. suggest that antibiotic resistance is common in children in Nairobi with severe malnutrition [35]. However, many of the isolates were not obtained from blood cultures; 8/21 of blood isolates were contaminants (including coagulase-negative staphylococci), which accounted for a large proportion of antibiotic resistance. Of interest, the authors also noted the limited scope of cefuroxime and chloramphenicol for the gram-negative isolates, but noted the potential usefulness of ciprofloxacin. Our data strongly support consideration of cheaper fluoroquinolones (e.g., norfloxacin or ciprofloxacin) as an addition to the current regime or as a second-line therapy to prevent late deaths. However, further studies are required to identify the population at risk and, owing to the expense of intravenous formulations, to determine whether the pharmacokinetics of oral preparations suggest that they could be used in this situation. Moreover, although aminoglycosides are the mainstay of treatment for gram-negative sepsis, we demonstrate in this current series an important association of gram-negative infection with early fatality. The deaths of children on adequate antimicrobial treatment point to the need for pharmacokinetic studies [36] examining the adequacy of once-daily 7.5-mg/kg gentamicin dosing. This information would also provide important reference data on the use of the recommended antibiotic policy in situations without routine therapeutic drug monitoring.

In summary, we have identified a number of readily discerned clinical features that could define a high-risk group to target emergency treatment and to rationalize limited resources in the context of a busy paediatric ward in Africa. Nevertheless, these will need formal assessment together with more basic research to identify targeted simple treatments, which may enhance the operational feasibility, cost effectiveness, and success of current malnutrition protocols.

## Abbreviations

CI: confidence interval
CRT: capillary refill time
Hb: haemoglobin
LR: likelihood ratio
MUAC: mid–upper arm circumference
OR: odds ratio
WHO: World Health Organization
WHZ: weight-for-height z-score
